# Focal cortical thickness correlates of exceptional memory training in Vedic priests

**DOI:** 10.3389/fnhum.2014.00833

**Published:** 2014-10-20

**Authors:** Giridhar P. Kalamangalam, Timothy M. Ellmore

**Affiliations:** ^1^Department of Neurology, The University of Texas Health Science Center at HoustonHouston, TX, USA; ^2^Department of Psychology, The City College of New YorkNew York, NY, USA

**Keywords:** MRI, prefrontal cortex, temporal lobe, hippocampus, hemispheric encoding and retrieval asymmetry (HERA)

## Abstract

The capacity for semantic memory—the ability to acquire and store knowledge of the world—is highly developed in the human brain. In particular, semantic memory assimilated through an auditory route may be a uniquely human capacity. One method of obtaining neurobiological insight into memory mechanisms is through the study of experts. In this work, we study a group of Hindu Vedic priests, whose religious training requires the memorization of vast tracts of scriptural texts through an oral tradition, recalled spontaneously during a lifetime of subsequent spiritual practice. We demonstrate focal increases of cortical thickness in regions of the left prefrontal lobe and right temporal lobe in Vedic priests, in comparison to a group of matched controls. The findings are relevant to current hypotheses regarding cognitive processes underlying storage and recall of long-term declarative memory.

## Introduction

Identifying the neuroanatomical correlates of skill acquisition in special populations remains a fruitful strategy in cognitive science research (Zatorre et al., 2012; Gu and Kanai, 2014). The iconic London taxi driver study (Maguire et al., 2000), for example, highlighted the role of the right posterior hippocampus in human spatial memory. There is now a large literature on the neuroimaging of human learning (Poeppel and Krause, 2008; Fields, 2011) that parallels the evolving conceptualization of brain memory systems (Eustache and Desgranges, 2008; Squire and Wixted, 2011; Maguire, 2014). In general, imaging of cognition attempts to connect a putative brain region or network to the cortical function under study, using metrics defined by structural MRI (e.g., gray matter volume, white matter tract density), functional-metabolic methods (e.g., functional MRI (fMRI) activations, positron emission tomography maps), or connectivity analysis of dynamic data (e.g., resting state fMRI, electroencephalography). When these modalities are used in combination, concordance of the results may be used to strengthen the biological hypotheses under consideration (Hermundstad et al., 2013; Fauvel et al., 2014). Nevertheless, structural (“fixed”) brain change related to cognitive activity implies reorganization of local neural architecture over a much longer time scale than, for instance, task-associated fMRI-detected blood flow change. Thus, structural imaging may be applied to probe cognition in populations with particular expertise—those who repeatedly recruit specific cortical areas over time scales of weeks, months or years (Maguire et al., 2000; Gaser and Schlaug, 2003; Jancke et al., 2009; Halwani et al., 2011).

In this work, we demonstrate discrete foci of increased cortical thickness in a group of Hindu Vedic priests in comparison to matched controls. Vedic priests are a unique and highly-specialized cohort whose scriptural education mandates extraordinary memory training over several years. We were interested in identifying structural correlates of such cognitive activity. We hypothesized that alterations in gray matter thickness would be observed in cortical regions participating in the encoding, storage and retrieval of verbal semantic and prosodic material.

## Methods

Eleven male right-handed Vedic priests in the age range 21–45 years (mean 33.3) consented to the study. All were in perfect health, on no medications, and had no previous history of significant illness. Eleven healthy male right-handed volunteers, all with college degrees and matched for age (mean 32.6 years, range 24–50), served as the control group. Priests had received 8–12 years of training (mean 9.45), starting at age 5–18 (median 10). All were in active priestly practice; the median number of years elapsed from the time of completion of training was 11 (range 5–30).

Magnetic resonance imaging (MRI) volumes (3T Philips Intera scanner, 8-channel head coil, SENSE acquisition, T1-weighted magnetization-prepared turbo field echo sequence, inversion time 1141.5 ms, shot interval 3000 ms, acquisition bandwidth 177 Hz/pixel, TR/TE = 8.4/3.9 ms; flip angle = 8°; matrix size = 256 × 256; field-of-view = 240 mm; slice thickness = 1.0 mm) were acquired from each subject in a single imaging session.

All scans were manually reviewed to identify excessive movement, gradient non-linearity and B_1_ field inhomogeneity artifact. Data processing was carried out in FREESURFER v5.3.0 (Dale et al., 1999; Fischl et al., 1999). The software generates a surface representation of the cortex as a meshwork of vertices from the high-resolution anatomical MRI volume, delineating the gray-white interface and measuring the distance across the cortical mantle (i.e., cortical thickness) at each vertex point. These processing steps yielded measures of total cortical gray matter volume (TCGMV) and mean cortical thickness of the left and right hemispheres (MCTL, MCTR respectively) for each subject. Group comparisons between priests and non-priests of TCV and MCT were carried out with independent-samples *t*-tests. Cortical thickness was visualized by mapping vertex-wise thickness values on to individual cortical surfaces, with group comparison performed with a processing stream implicit in FREESURFER. Thickness data from all subjects were sampled to FREESURFER's common *fsaverage* space and smoothed with a symmetric Gaussian kernel of full-width half-maximum (FWHM) 10 mm. Statistically significant regional thickness differences between the groups were investigated with a general linear model of the effect of profession (i.e., priest vs. non-priest) at each surface vertex. Correction for multiple comparisons was performed by a cluster-based method (Hagler et al., 2006) that generates random clusters from smoothed maps of Gaussian noise with cluster size limits generated through a Monte Carlo simulation. The process was iterated 10,000 times; clusters in the real data were compared with the random distribution and those with cluster-wise *p*-value (CWP) < 0.05 retained. Finally, the retained clusters were resampled in the reverse direction from *fsaverage* onto all 22 individual surfaces to obtain subject-wise summary statistics of the group significance maps.

The study was approved by the Institutional Review Board of the University of Texas Health Science Center-Houston.

## Results

TCGMV and MCTL/R raw data for all 22 subjects appear in Table 1. There were no significant differences in TCGMV, MCTL, and MCTR between the priest and non-priest group (Student's *t*-test for independent samples, *p* = 0.32, *t* statistic = 1.036, d.o.f = 10; *p* = 0.61, *t* statistic = −0.529, d.o.f = 10; *p* = 0.75, *t* statistic = −0.3288, d.o.f = 10, respectively).

**Table 1:**
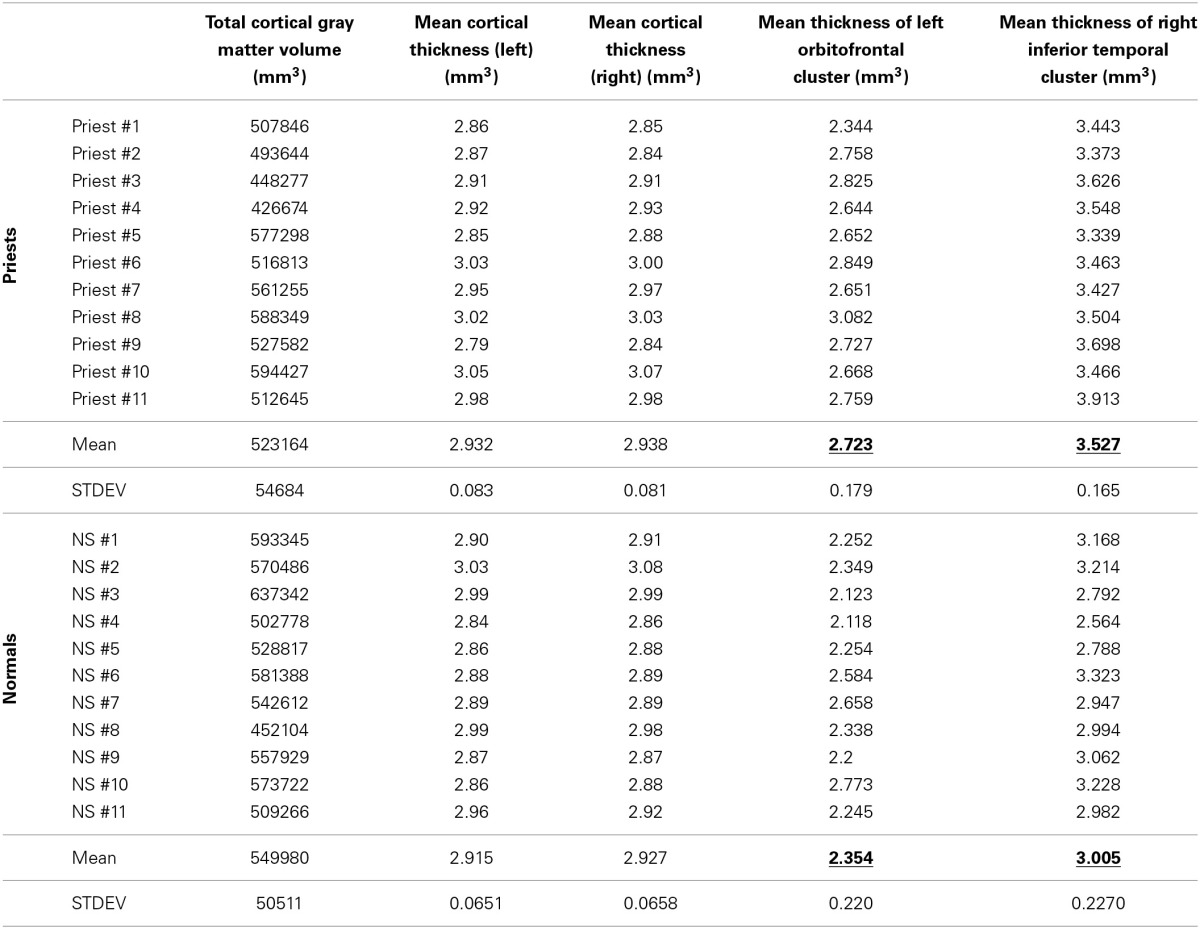
**Total cortical gray matter volume, left and right mean cortical thickness, and thickness data of significant clusters for all 22 subjects**.

*There were no systematic differences between the two groups for the first three measures. The priest group however exhibited appreciably larger mean thickness in the two cluster locations that were confirmed statistically significant (underlined bold numbers; see text). STDEV, standard deviation; NS, normal (control) subject*.

**Figure 1:**
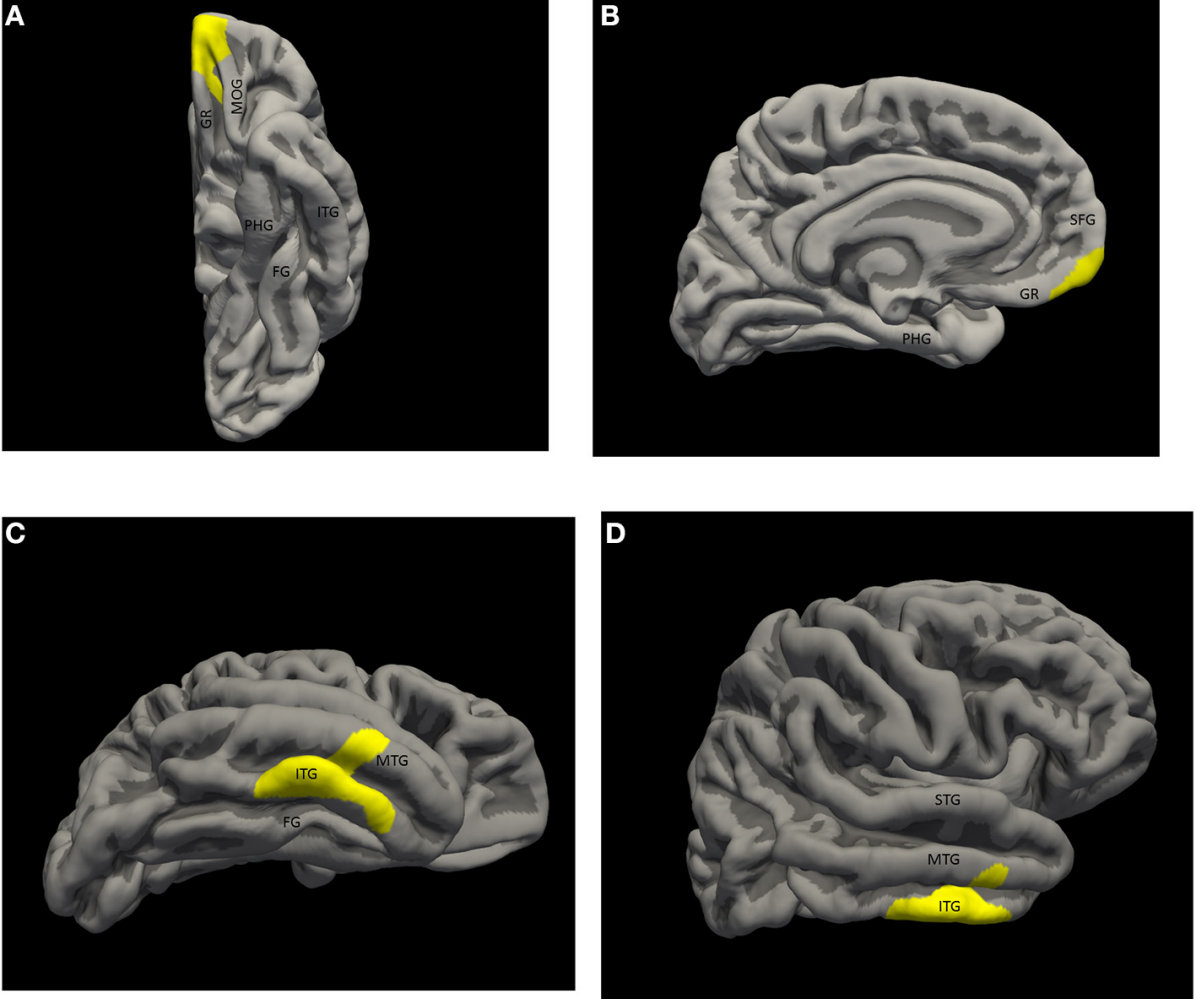
**Significant thickness clusters displayed over the pial surface reconstruction of FREESURFER's average template brain. (A,B)** Inferior and medial views of the left hemisphere, show a single cluster extending over the medial, ventral and polar orbito-frontal cortex. **(C,D)** Inferior and lateral views of the right hemisphere show a larger cluster occupying the middle inferior temporal neocortex, over the inferior temporal gyrus, the inferior temporal sulcus and part of the middle temporal gyrus. Key: STG, superior temporal gyrus; MTG, middle temporal gyrus; ITG, inferior temporal gyrus; FG, fusiform gyrus; PHG, parahippocampal gyrus; SFG, superior frontal gyrus; GR, gyrus rectus; MOG, medial orbital gyrus.

There were two statistically significant clusters of focal cortical thickening in the priest group compared to the control group (Figures 1A–D). The first was in the left orbitofrontal cortex including the anterior portion of the gyrus rectus and medial orbital gyrus (Brodmann areas 10/11/14, cluster size 460 mm^2^, MNI coordinates at peak vertex = [−9.5, 52.6, −21.8], *p* < 0.001 at peak vertex, cluster-wise *p* = 0.027 corrected for multiple comparisons). The second was over the right inferior temporal gyrus and middle temporal gyrus, straddling the inferior temporal sulcus (Brodmann areas 20/21, cluster size 601 mm^2^, MNI coordinates at peak vertex = [53.0, −17.5, −28.4], *p* < 0.001 at peak vertex, cluster-wise *p* = 0.008 corrected for multiple comparisons). The mean thicknesses of these two areas in individual subjects are listed in Table 1; significantly higher values in the priest group are suggested on even casual inspection.

There were no significant clusters in the opposite sense, i.e., focal areas of significant relative cortical thinning in the priest group.

## Discussion

This work was motivated by the singular characteristics of our study population. Vedic priests are required to master spiritual textual material (extracts from the *Vedas* and their ancillary texts), most of which were composed prior to the invention of writing (Scharfe, 2002). The core of Vedic education is rote memorization of scriptural hymns in the classical Sanskrit language, stanza-by-stanza, by oral discourse from teacher to student. This method of instruction has remarkably stayed true to its origin in the early Vedic period (~1000 BC) (Altekar, 1944). Scripture memorization is achieved by repeated recitation of short hymn segments set to a characteristic cadence and melodic contour (Howard, 1986). During a full-time apprenticeship lasting 8–12 years (spanning childhood through early adulthood), a prospective Vedic priest may memorize in excess of 10,000 stanzas in this fashion (at 32 syllables per stanza, the material in written form typically occupies over a thousand printed pages; uninterrupted recitation of it all at normal articulatory speeds—200 stanzas per hour—would take over two continuous days and nights). To our knowledge, the assimilation of auditory-verbal material of this quantity in a specialized cohort is unsurpassed. Such an exceptional cognitive achievement, we surmised, would be reflected in differences in brain structure between priests and normal subjects.

It is well-established that the medial temporal lobes in humans are critical to the formation of long-term explicit (declarative) memory (Squire and Wixted, 2011). Following an initial period of consolidation (Squire and Bayley, 2007), memories disengage from the hippocampus and distribute themselves over multiple neocortical locations. The continuing role of the hippocampus in the retrieval (Winocur et al., 2010) and reconsolidation (Tronson and Taylor, 2007) of remote memory is under debate, though long-term memories of purely semantic nature are considered hippocampus-independent (Levy et al., 2004; Winocur and Moscovitch, 2011). The sites of neocortical storage appear to be those that were initially deployed the processing of the memory (Squire and Wixted, 2011); indeed, the process of recall of multi-sensory experience reactivates the very areas that were activated during encoding (Danker and Anderson, 2010). On this basis, we expected significant structural changes in priest brain areas important for verbal semantic encoding and retrieval—the language dominant prefrontal cortex, according to the hemispheric asymmetry and retrieval activation model (HERA) (Tulving et al., 1994; Habib et al., 2003). Further, due to the importance of tone and meter to process of Vedic scriptural memorization, we surmised that auditory areas in the language non-dominant temporal lobe might show thickness changes as well (Peretz and Zatorre, 2005). Due to the right-handedness of all our subjects, we viewed the left hemisphere as language-dominant in all individuals, a strong but not unreasonable assumption (Knecht et al., 2000). Finally, given the long-term semantic nature of the learned material, we hypothesized that hippocampal changes would be absent. Our observations were broadly consistent with these expectations.

Several functional imaging studies have confirmed the HERA model, establishing that the prefrontal cortex participates in memory formation, with the type of memory (verbal or non-verbal) determining the laterality (left or right, respectively) of activation (Tulving et al., 1994; Kapur et al., 1995; Nyberg et al., 1996; Buckner et al., 1999). Controlled retrieval of long-term verbal memory engages prefrontal cortex (Danker et al., 2008), with the frontal pole and ventro-inferior prefrontal cortex activated in a material-specific manner in both working memory and long-term memory paradigms (Braver et al., 2001). One view of the organization of the prefrontal cortex is of a dorsal-ventral and rostro-caudal hierarchy, with the more rostral and ventral areas concerned with increasingly higher-order retrieval coordinative tasks, such as controlling retrieval in accordance to context, or integrating retrieval across large time scales (Race et al., 2009). The left frontal thickening seen in our priest population was entirely ventral, extending rostrally to the frontal pole. Thus, these findings were consistent with the habitual use of the brain areas involved in verbal encoding and controlled, context-dependent retrieval. Interestingly, there is evidence from a study of musical memory (Platel, 2005) that prefrontal fMRI activation in an experiment testing retrieval of semantic musical memory (as opposed to episodic musical memory) is orbitofrontal and medial frontal in location (rather than ventro-lateral). The former location is more concordant with our results, and may relate to the peculiarity of the verbal semantic processing in Vedic training—its association with meter and tone.

The right temporal lobe cortical thickening in priests may be understood by considering the processing of acoustic verbal information. Numerous functional imaging paradigms have established that the spoken word activates the superior temporal gyrus (STG) bilaterally (Hickok, 2009). According to the “dual stream” hypothesis (Hickok and Poeppel, 2007), subsequent phonological processing then proceeds in a left-dominant “dorsal stream” comprising the posterior superior temporal lobe, parietal operculum and posterior frontal lobe, and a bilaterally represented “ventral stream” that projects into anterior and middle temporal lobe areas. Phonological processing in the left and right ventral streams is thought to be computationally asymmetric, with the left hemisphere “sampling” information at a higher rate than the right hemisphere (Boemio et al., 2005). An alternative view is that the left hemisphere computes in the time domain, and the right hemisphere in the frequency domain (Zatorre et al., 2002). These ideas seek to explain the left-hemispheric dependence of high-frequency phonological (syllabic) information discrimination from the right-hemispheric slower frequency (prosodic) discrimination (Poeppel et al., 2004). Despite computational differences, both hemispheres are thought to process phonological information sufficiently for semantic-lexical access. Assuming that auditory speech information with strong prosodic content—as encountered in Vedic training—preferentially engages right temporal lobe mechanisms, the right temporal neocortex would be the main repository for those semantic memories (Binder and Desai, 2011; Squire and Wixted, 2011). On a different point, there is evidence that setting verbal material to melody and meter facilitates its memorization (Purnell-Webb and Speelman, 2008), and the strategy of associating text with cadence and tone may have historically evolved as a cognitive tool in Vedic education.

It is instructive to compare our results with other structural imaging observations of human memory. In the study of Maguire and colleagues (Maguire et al., 2003) a group of 10 individuals of exceptional memory ability were compared to a group of matched controls. No systematic differences between the groups were found on gray matter volume measurements by voxel-based morphometry. Instead, the authors found on functional imaging experiments that the superior ability of exceptional memorizers was based on novel spatial cognitive strategies. Given the unequivocal gray matter thickness changes in our priest group, we conclude that—as hypothesized—the changes seen were not a reflection of innate talent, but instead were a consequence of memory training. These conclusions are also supported by the lack of population bias (Bavelier et al., 2009) in our study: children who undergo Vedic training do so as a family tradition in certain Hindu families and are not chosen for any special abilities beyond a basic capacity for education. All priests in our cohort also received, as is usual, a few years of normal schooling prior to entering Vedic apprenticeship. On the other hand, our results are highly concordant with a recent study (Engvig et al., 2010) on the effects of memory training on cortical thickness in a group of upper middle-aged subjects. In this study, 22 healthy subjects were administered an 8-week intensive memory training program consisting of word list memorization through a spatial visualization mnemonic method. The latter method—in effect, a cognitive tool—links the to-be-remembered words with a spatial route of various landmarks during encoding. At recollection, the route is remembered, together with the associated words. Much as with the link to rhythm and melody, the linkage of verbal material to visuospatial imagery has been shown to facilitate memorization (Lindenberger et al., 1992). The authors found cortical thickness increase over both orbito-frontal cortices, the right fusiform gyrus, and the right insula. From discussion above, we presume the left prefrontal changes were related to verbal encoding and retrieval, with the homologous changes on the right due to the strongly visuospatial nature of the encoding and recall process. Interestingly, the right fusiform gyrus thickness change observed by these authors co-localized precisely with lesion location in patients with so-called topographic landmark amnesia (Aguirre and D'Esposito, 1999), wherein patients lose the ability to mentally navigate among landmarks, though they have no difficulty is recognizing the landmarks themselves. Presumably, the cortical thickening seen in the right fusiform gyrus by the authors reflected the activity of this cortical area in binding verbal memory to landmarks and the storage of the verbal-spatial memory complex. The right insular changes were less definitive in origin, as the authors themselves imply. Tellingly, the extent of insular changes did not correlate with memory score improvements (contrasted to the prefrontal and temporal lobe changes). The authors speculate that the insular thickening reflected the role of that region in coordinating between brain states during the encoding process, and did not directly reflect processes associated with memory improvement. Our results (lack of significant insular changes in priests) would support these conclusions.

We comment on a few technical issues. Our choice of FREESURFER was based on the software's documented accuracy in measuring cortical thickness from T1-weighted MRI images (Fischl and Dale, 2000), and its validation by both histology (Rosas et al., 2002) and expert manual review (Kuperberg et al., 2003). We were content to acquire a single T1 volume, rather than acquiring multiple volumes and averaging as practiced by some authors. Our strategy was based on FREESURFER's documented insensitivity to T1-data redundancy in the absence of scanner and software upgrades (Han et al., 2006; Jovicich et al., 2009). The multiple-comparisons testing carried out in FREESURFER is not a Bonferroni-type *p*-value correction. Clusters are instead chosen by vertex-wise thresholds that the experimenter specifies (we specified *p* < 0.01), comparing cluster sizes with those generated randomly, and retaining cluster sizes specified through a second threshold (we chose *p* < 0.05). Thus, the “multiple comparison” aspect of the processing is essentially a cluster-wise thresholding procedure, and the method could fail to pick up small clusters of subthreshold size with high vertex-wise *p*-values. Our choice of a small FWHM (10 mm) for raw thickness data smoothing (see Methods) attempted to deal with this caveat. In other words, we consider our detection of significant clusters accurate down to a characteristic cluster length dimension of ~10 mm (or area ~100 mm^2^). Areas of significance much smaller than this would be neglected in our analysis, though equally we would have difficulty in ascribing specific functional significance to areas of such small size in the association cortex.

One weakness of our study was its unimodality: we were unable to perform additional structural analysis (e.g., white matter tractography) or any dynamic analyses (e.g., resting state fMRI connectivity). Another weakness was our modest sample size. It will be interest to extend our analyses in the future to a bigger group, specifically exploring structural and functional connectivity between the areas of increased cortical thickness. However, we believe our findings are significant for several reasons. Among primates, the capacity for long-term auditory memory may be uniquely human, and may relate to the human language ability (Fritz et al., 2005). Thus, our results demonstrate the first brain morphometric correlates of this skill, when highly developed. Second, the task-related changes pertained to a relatively narrow cognitive “bandwidth”: the material memorized by priests was entirely semantic and non-spatial in content, and largely absorbed through an auditory route. Our results therefore pertain to a “pure” cognitive paradigm, and may serve to benchmark related studies in the future. Third, our results are concordant with, and inform, an expansive literature in the neuroscience of memory, whether from the imaging, neuropsychological, or neurological lesion-deficit point of view. Particularly informative is our observation of “verbal” memory being effectively right-lateralized in the temporal lobe in right-handers due to its association with tone and meter. Finally, we demonstrate the enduring value of the study of expertise in highlighting brain mechanisms; and in a unique population never before studied in this manner.

Longitudinal imaging with more subjects will elucidate the finer details of cognitive processes involved in the process of Vedic priesthood training (e.g., the relative roles of the dominant and non-dominant hippocampi as the material is learned), and changes associated with later stoppage of priestly practice and aging. A larger study will also enable exploration of the individual roles of factors such as age of training onset, years of training, and duration of post-training practice on the imaging changes.

### Conflict of interest statement

The authors declare that the research was conducted in the absence of any commercial or financial relationships that could be construed as a potential conflict of interest.
